# The importance of CA 72-4 and CA 19-9 dosing in gastric cancer

**DOI:** 10.25122/jml-2022-0173

**Published:** 2023-02

**Authors:** Mihai Cătălin Roșu, Andrei Ardelean, Silviu Daniel Moldovan, Flaviu Ionut Faur, Alexandru Nesiu, Bogdan Dan Totoloci

**Affiliations:** 1 Department of General Surgery, Faculty of Medicine, Vasile Goldiș Western University of Arad, Arad, Romania; 2 Department of General Surgery, Arad County Emergency University Hospital, Arad, Romania; 3 Department of Urology, Arad County Emergency University Hospital, Arad, Romania; 4 2^nd^ General Surgery Clinic, Pius Brinzeu Emergency Clinical Hospital, Timisoara, Romania

**Keywords:** CA 72-4, CA 19-9, gastric cancer, tumour marker

## Abstract

Serological analysis of tumor markers has emerged as a non-invasive method for monitoring cancer patients, including tumor recurrence and response to treatment. Tumor markers have the potential to aid in both the diagnosis and prognosis of cancer, but their most important role currently lies in the monitoring of tumor progression. Tumor markers can also provide valuable information on treatment effectiveness, with changes in plasma values indicating tumor regression or progression. This research aimed to investigate the correlation between the serum detection values of three tumor markers – CEA, CA 19-9, and CA 72-4 - and their utility in the diagnosis and prognosis of patients with gastric cancer. The study seeks to uncover the relationship between these tumor markers and the evolution of gastric cancer, providing insights into their potential use in clinical practice.

## INTRODUCTION

Gastric cancer is a major health concern worldwide, and serum markers have been investigated for their potential utility in diagnosis, prognosis, and follow-up of patients. Studies have reported elevated serum levels of CEA and CA19-9 in a significant proportion of patients with gastric cancer, ranging from 15% to 72%. However, the use of these markers for follow-up has shown some limitations, indicating a need for more sensitive performance markers to enhance gastric cancer treatment [1-3]. The monoclonal antibodies B72.3 and CC49 have been used to characterize high molecular weight mucus protein, formerly known as tumor-associated glycoprotein-72 (TAG-72), as a potential marker for various types of cancer since 1986. In particular, elevated serum levels of CA 72-4 have been observed in a significant proportion of patients with gastrointestinal malignancies, and ovarian, endometrial, lung, and breast cancer [4-6]. Some studies have rated CA 72-4 as a serological tumor marker for gastric cancer and have compared its clinical utility with other markers such as CEA or CA19-9. Multiple studies have found that serum levels of CA 72-4 show a strong correlation with tumor stage and the presence of lymph node involvement in gastric carcinoma. As a result, measuring serum levels of CA 72-4 is not considered useful for early assessment, but it can provide valuable insights into the potential recurrence of cancer. While previous research has investigated the correlation between preoperative CEA and CA 19-9 concentration and prognosis, there are limited reports on the prognostic value of CA 72-4 in gastric cancer [7,8].

In this literature review, we explored the current understanding of serum markers for gastric cancer, with a particular focus on CA72-4, CEA, and CA19-9. We reviewed the literature related to their diagnostic accuracy, sensitivity, and specificity, as well as their potential for use in prognosis and follow-up. We also considered and compared the sensitivity of CA72-4 in different gastric cancer disorders and compared it to the sensitivity of CEA or CA19-9. By synthesizing the existing literature, we aimed to provide an overview of the current state of knowledge and identify areas for future research.

## MATERIAL AND METHODS

To achieve the objectives of this study, a systematic search of the PubMed database was conducted. The search was limited to articles published between March 1st, 2015, and May 2022, and included the following keywords: "CEA", "CA 72-4", and "CA 19-9". Only articles written in English were evaluated. The final set of articles included in this study underwent a thorough review and analysis to provide a comprehensive overview of the current understanding of the sensitivity and prognostic value of these markers in the context of gastric cancer using a descriptive analysis.

## RESULTS

### CA72-4 as a prognostic marker

Despite the multitude of markers that have been identified, CA 72-4 is of particular interest. Some preliminary studies have shown that CA 72-4 has elevated levels mainly in patients with gastrointestinal and ovarian cancer, while low values have been documented in patients with other types of cancer, such as breast, prostate, and lung cancer [9,10]. Numerous other studies have outlined the clinical importance of the serological marker CA 72-4 in terms of the management of patients with gastric and gynecological cancer. Some preliminary studies have suggested that the presence of serum CA 72-4 was detected in 40% of patients with gastrointestinal adenocarcinoma, while subsequent reports focused on exploring potential correlations between CA 72-4 and other markers such as CEA and CA 19-9 [11,12].

### CA19-9 as a prognostic marker

CA19-9 is an oligosaccharide present in both tissues and serum in the form of mucin-rich carbohydrates. It was first isolated in 1979 from colorectal carcinoma (Kaprowski) and is normally found in the fetal cells from the stomach, the intestine, the liver, and the pancreas. In adults, it is present in small amounts in the pancreas, the liver, the gallbladder, and the lungs and is an important component of many mucous cells and secretion products. CA 19-9 is used predominantly for the prognosis of pancreatic adenocarcinoma, but it is not a specific biomarker, its expression is also used in gastric cancer. High levels of CA 19-9 depend on the staging degree of gastric cancer, as shown by numerous studies in Japan [13,14]. In the later stages of gastric cancer, CA 19-9 can be useful for both prediction and diagnosis. A recent study found that only 4.8% of patients who underwent radical (total) gastrectomy had elevated CA 19-9 values. Another study conducted in India reported a sensitivity of 42% and a negative predictive value of 63% for the CA 19-9 test in gastric cancer. A separate study involving 1,600 gastric cancer patients who underwent gastrectomy found that CA 19-9, CA 125, and CEA positivity rates were 20%, 42.3%, and 19.2%, respectively. The researchers also observed that CA 19-9 levels tended to increase more significantly in older and female patients compared to younger and male patients [15,16].

### CA72-4 as a prognostic marker

CA 72-4 was first identified by Colcher in 1981 and has since been identified as a tumor-associated glycoprotein-72 antigen (TAG-72) in several epithelial cancers. Several recent studies have demonstrated the usefulness of CA 72-4 in the diagnosis and prognosis of gastric cancer. One study found that serum levels of TAG-72 expressed as CA 72-4 have a significant impact on the diagnosis of gastric cancer [17]. An Italian longitudinal study with over 160 patients has highlighted the importance of monitoring serum levels of CA 72-4 in gastric cancer. The study demonstrated that CA 72-4 is an independent marker that can be used for the prognosis and assessment of recurrences in gastric cancer [10]. The results showed that nearly half of the patients with recurrent gastric cancer had elevated serum levels of CA 72-4 before surgical interventions, compared to approximately 24 percent of patients without recurrence. These findings suggest that monitoring CA 72-4 levels could be valuable in detecting early signs of recurrence in gastric cancer patients. One French study reported that serum levels of CA 72-4 were associated with a poor prognosis in male patients with gastric cancer, even when their CA 19-9 and CEA levels were within the normal range before the start of treatment [17].

In another recent study, 216 patients with gastric adenocarcinoma were examined to evaluate the serum levels of CEA, CA 19-9, and CA 72-4. The findings showed no significant differences in the values of CA 72-4, CEA, or CA 19-9 with regard to sex, age, or histological classification [18].

### Other markers associated with gastric cancer stem cells

Recent studies have suggested a potential association between markers typically associated with gastric cancer stem cells, such as Lgr5 and Dclk1, and other cancer markers [19]. For example, studies have shown that Lgr5 is overexpressed in gastric cancer and may play a role in the development and progression of the disease [19]. Similarly, Dclk1 is upregulated in gastric cancer and may also play a role in the growth and spread of the disease [19]. However, more research is needed to fully understand the connections between these markers and the role they play in gastric cancer development.

Lgr5 (Leucine-rich repeat-containing G protein-coupled receptor 5) is a transmembrane protein that is considered a marker for cancer stem cells (CSCs) in various types of cancer, including gastric cancer. Lgr5 is a member of the G protein-coupled receptor (GPCR) family and is involved in signaling pathways related to cell growth, differentiation, and survival. Studies have shown that Lgr5 is overexpressed in gastric cancer and may play a role in the development and progression of the disease [19]. It has been found that Lgr5+ cells can initiate and maintain tumors, and they are resistant to chemotherapy, making Lgr5 a promising therapeutic target for gastric cancer treatment. Therefore, Lgr5 may provide a potential target for developing new therapies for gastric cancer, as well as a diagnostic marker for identifying CSCs in gastric cancer [19].

Dclk1 (doublecortin-like kinase 1) is a protein kinase that has been identified as a marker for cancer stem cells (CSCs) in various types of cancer, including gastric cancer. It is a member of the doublecortin-like kinase (DCLK) family, which is involved in the regulation of cell division, migration, and differentiation [19]. Studies have shown that Dclk1 is upregulated in gastric cancer and may play a role in the growth and spread of the disease[19]. Dclk1+ cells have been found to have higher tumorigenic and metastatic potential than Dclk1- cells. Furthermore, Dclk1+ cells are more resistant to chemotherapy and radiation, making Dclk1 a promising therapeutic target for gastric cancer treatment. Therefore, Dclk1 may provide a potential target for developing new therapies for gastric cancer, as well as a diagnostic marker for identifying CSCs in gastric cancer [19].

## DISCUSSION

In this study, we aimed to evaluate the diagnostic and prognostic value of CEA, CA 19-9, and CA 72-4 in gastric cancer. Previous research has shown that elevated serum CEA levels are frequently predictive of advanced gastrointestinal carcinoma and are correlated with it [18]. In addition, a persistent postoperative increase in CEA may signal the need for a second opinion procedure and the potential existence of local recurrence or metastasis [18]. It is important to note, however, that not all patients with GI adenocarcinoma display positive preoperative or increasing postoperative serum CEA levels, and some do not experience disease recurrence [18].

Compared to isolated biomarkers 72-4, combinations in GC patients show less co-presentation of CEA, CA 19-9, and CA [18]. The combination of CEA, CA 19-9, and CA 72-4 is the most efficient option for staging surgery or chemotherapy for GC patients, according to the findings of this review. Higher sensitivity and specificity were demonstrated by the simultaneous detection of serum CEA, CA 19-9, CA 24-2, and CA 72-4 in people with GC and cardiac cancer [18]. The specificity may be improved by routinely measuring the levels of serum CA 19-9, CA 72-4, and CEA at suitable intervals.

However, it is important to note that this study has several limitations. The participant characteristics in the included studies varied, which could have affected the diagnostic utility of CA 19-9, CA 72-4, and CEA. Stratified analysis based on patient traits like blood type was not carried out due to the lack of available data. Additionally, the source of heterogeneity among the included studies was incomplete, as studies and patient characteristics were infrequently reported. Finally, publication bias is a problem that cannot be avoided in meta-analyses of published studies.

To confirm the clinical importance of serum CA 72-4, CA 19-9, and CEA in gastric cancer, large prospective studies are required. These studies should focus on addressing the limitations of previous research, including controlling for patient characteristics, reporting complete study details, and avoiding publication bias.

## CONCLUSION

The use of serum markers like CA 72-4 and CA 19-9, in addition to CEA, can aid in the diagnosis and monitoring of advanced gastric cancer, especially in cases where CEA levels are not detectable. Simultaneous measurement of these markers can improve the precision of treatment options like chemotherapy or second eye surgery. While these markers are not useful for screening early gastric cancer, they are important in detecting recurrent metastases and for post-therapeutic follow-up. However, careful consideration should be given to the combined measurements of CEA, CA 19-9, and CA 72-4, particularly when the values are at the limit or not significantly elevated. Further studies are needed to fully understand the clinical importance of these serum markers in gastric cancer and to develop more effective diagnostic and treatment strategies.

## References

[1] Liang Y, Wang W, Fang C, Raj SS (2016). Clinical significance and diagnostic value of serum CEA, CA19-9 and CA72-4 in patients with gastric cancer. Oncotarget.

[2] Yang AP, Liu J, Lei HY, Zhang QW (2014). CA72-4 combined with CEA, CA125 and CAl9-9 improves the sensitivity for the early diagnosis of gastric cancer. Clin Chim Acta.

[3] Yu J, Zhang S, Zhao B (2016). Differences and correlation of serum CEA, CA19-9 and CA72-4 in gastric cancer. Mol Clin Oncol.

[4] Yin LK, Sun XQ, Mou DZ (2015). Value of combined detection of serum CEA, CA72-4, CA19-9 and TSGF in the diagnosis of gastric cancer. Asian Pac J Cancer Prev.

[5] Korse CM, Taal BG, Bonfrer JM, Vincent A (2011). An elevated progastrin-releasing peptide level in patients with well-differentiated neuroendocrine tumours indicates a primary tumour in the lung and predicts a shorter survival. Ann Oncol.

[6] Molina R, Bosch X, Auge JM, Filella X (2012). Utility of serum tumor markers as an aid in the differential diagnosis of patients with clinical suspicion of cancer and in patients with cancer of unknown primary site. Tumour Biol.

[7] Molina R, Auge JM, Alicarte J, Filella X (2004). Pro-gastrin-releasing peptide in patients with benign and malignant disease. Tumour Biol.

[8] Guadagni F, Roselli M, Cosimelli M, Mannella E (1993). TAG-72 (CA 72-4 assay) as a complementary serum tumor antigen to CEA in monitoring patients with colorectal cancer. Cancer.

[9] Guadagni F, Roselli M, Cosimelli M, Ferroni P (1995). CA 72-4 serum marker--a new tool in the management of carcinoma patients. Cancer Invest.

[10] Yu J, Zhang S, Zhao B (2016). Differences and correlation of serum CEA, CA19-9 and CA72-4 in gastric cancer. Mol Clin Oncol.

[11] Gero EJ, Colcher D, Ferroni P, Melsheimer R (1989). CA 72-4 radioimmunoassay for the detection of the TAG-72 carcinoma-associated antigen in serum of patients. J Clin Lab Anal.

[12] Ferroni P, Szpak C, Greiner JW, Simpson JF (1990). CA 72-4 radioimmunoassay in the diagnosis of malignant effusions. Comparison of various tumor markers. Int J Cancer.

[13] Ohuchi N, Takahashi K, Matoba N, Sato T (1989). Comparison of serum assays for TAG-72, CA19-9 and CEA in gastrointestinal carcinoma patients. Jpn J Clin Oncol.

[14] Ychou M, Duffour J, Kramar A, Gourgou S, Grenier J (2000). Clinical significance and prognostic value of CA72-4 compared with CEA and CA19-9 in patients with gastric cancer. Dis Markers.

[15] Chen XZ, Zhang WK, Yang K, Wang LL (2012). Correlation between serum CA724 and gastric cancer: multiple analyses based on Chinese population. Mol Biol Rep.

[16] Jing JX, Wang Y, Xu XQ, Sun T (2014). Tumor markers for diagnosis, monitoring of recurrence and prognosis in patients with upper gastrointestinal tract cancer. Asian Pac J Cancer Prev.

[17] Guadagni F, Roselli M, Cosimelli M, Ferroni P (1993). Correlation between positive CA 72-4 serum levels and lymph node involvement in patients with gastric carcinoma. Anticancer Res.

[18] Takahashi Y, Takeuchi T, Sakamoto J, Touge T (2003). The usefulness of CEA and/or CA19-9 in monitoring for recurrence in gastric cancer patients: a prospective clinical study. Gastric Cancer.

[19] Wu XS, Xi HQ, Chen L (2012). Lgr5 is a potential marker of colorectal carcinoma stem cells that correlates with patient survival. World J Surg Oncol.

